# *In vitro* anticancer potentiality and molecular modelling study of novel amino acid derivatives based on *N*^1^,*N*^3^-bis-(1-hydrazinyl-1-oxopropan-2-yl) isophthalamide

**DOI:** 10.1080/14756366.2019.1613390

**Published:** 2019-07-09

**Authors:** Asmaa F. Kassem, Gaber O. Moustafa, Eman S. Nossier, Hemat S. Khalaf, Marwa M. Mounier, Suliman A. Al-Yousef, Sabry Y. Mahmoud

**Affiliations:** a Department of Chemistry of Natural and Microbial Products, Pharmaceutical and Drug Industries Research Division, National Research Centre, Giza, Egypt;; b Department of Peptide Chemistry, Chemical Industries Research Division, National Research Centre, Cairo, Egypt;; c Department of Pharmaceutical Medicinal Chemistry, Faculty of Pharmacy (Girls), Al-Azhar University, Cairo, Egypt;; d Department of Chemistry, College of Science and Arts, Jouf University, Al Qurayat, Saudi Arabia;; e Chemical Industries Research Division, Department of Photochemistry, National Research Centre, Cairo, Egypt;; f Pharmaceutical and Drug Industries Research Division, Department of Pharmacognosy, National Research Centre, Giza, Egypt;; g Department of Clinical Laboratory Science, College of Applied Medical Science, University of Hafr Al Batin, Hafr Al Batin, Saudi Arabia;; h Department of Biology, College of Science, University of Hafr Al Batin, Hafr Al Batin, Saudi Arabia

**Keywords:** Isophthalamide, amino acids, peptides, anticancer activity, molecular docking

## Abstract

A series of *N^1^,N^3^-bis (1-oxopropan-2-yl) isophthalamide*-based derivatives **4**–**16** were prepared and their structures were confirmed by different spectral tools. The cytotoxic potentiality of novel compounds **4**–**16** was assessed by the MTT assay method on colon, lung and breast tumour cell lines. Compound **5** gave the most significant specificity anticancer activity with safety response on normal cell lines. *In vitro* enzyme assay and several apoptotic parameters were examined to elucidate the mode of action of compound **5**. Molecular docking studies also were simulated to put insight and give better understanding to its structural features.

## 1. Introduction

Cancer is still the major health issue in most parts of the world and the prominent cause of death world-wide^1^. According to World Health Organization, cancer is the second leading cause of death and was responsible for 8.8 million deaths in 2015^2^. Cancer treatment has been the first goal of pharmaceutical industries over the last many decades^3^. Among the several treatment regimens available today, chemotherapy is most commonly used for treating many kinds of cancers world-wide. Drug resistance and failure of anti-tumour drugs are the main side effects and obstacles observed in successful development of anticancer drugs. Therefore, there is a vital need for the discovery of molecules which are endowed with excellent anticancer potency and lower side effects^4^^,^^5^.

**Figure 1.**
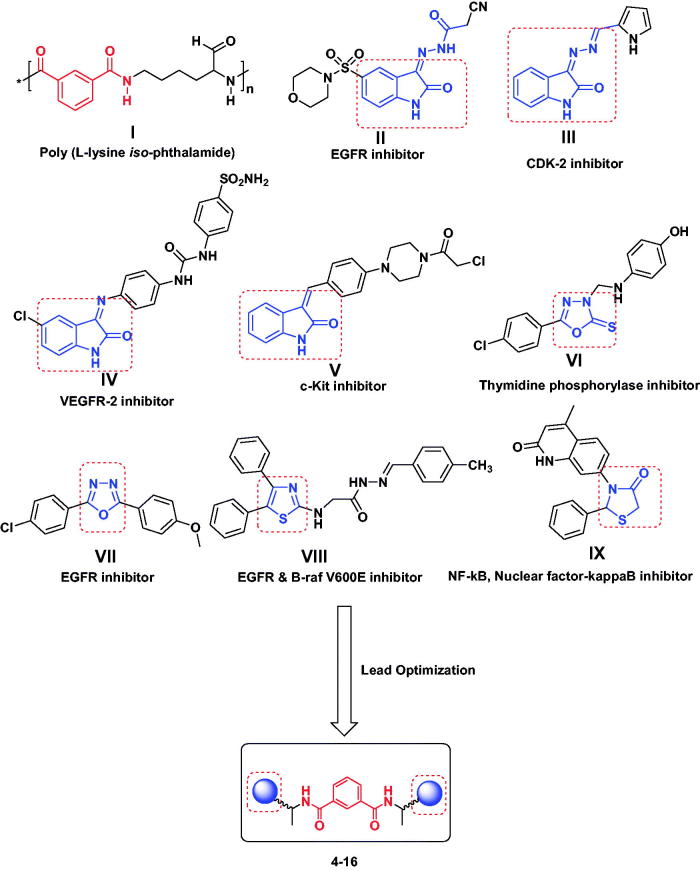
Design of newly synthesized isophthalamide based derivatives **4**–**16** concerning the chemical structures of poly (l-lysine isophthalamide) (**I**) and different heterocyclic motifs **II**–**IX** with various mechanisms of anticancer activity.

In the past years, biodegradable and biocompatible polymers have become an attractive choice as drug delivery systems. Biopolymers or biopeptides are an important class of low toxicity drug delivery vectors and their role has been long recognized with controlled release of drugs over long periods, easy conjugation for active targeting and adjustable release of both hydrophobic and hydrophilic molecules^6–8^. Amongst this class of materials, amphiphilic polymers can successfully transport a variety of molecules across mammalian cell membranes, such as cancer therapy candidates and proteins^9^^,^^10^. The transport of these polymers along different drugs to a particular site within cells has shown a great improvement in the efficacy and accessibility of therapeutics^11^^,^^12^. The parent polyamide, poly (l-lysine isophthalamide) (**I**) (Figure 1), was grafted with hydrophobic amino acids onto its pendant carboxylic acid groups to manipulate its amphiphilicity and structure^13^. Moreover, peptides comprise a major class of important anticancer therapeutic agents^14–16^.

Heterocyclic rings have chemical structural similarity with respect to the biologically active compounds within our body, such as DNA, RNA, neurotransmitters and hormones, so they become pivotal parts of bioactive molecules^17^. Heterocycles containing 2-oxoindoline, 1,3,4-oxadiazole, and thiazole scaffolds **II**–**IX** (Figure 1) exhibit significant antitumor activities via versatile and different mechanisms^18–25^. So, a combination of small heterocyclic molecules with a peptide motif (isophthalamide) can augment pharmacokinetic properties of both the peptide and the small molecules and provides more effective drug probes.

Depending on the previous observations and our program in the development of peptide fragments with anticancer activity^26–28^, some isophthalamide based derivatives were synthesized and evaluated for their cytotoxicity against human colon (HCT-116), lung (A-549) and breast (MCF-7) cancerous cell lines. The possible mechanisms of cytotoxicity action of the most active compound were further studied through *in vitro* enzymatic assay, DNA fragmentation and analysis of key proteins involved in the apoptotic pathways.

## 2. Experimental

### 2.1. Chemistry

Melting points were determined in an “Electro Thermal” Digital melting point apparatus (Shimadzu, Tokyo, Japan) (model: IA9100). Elemental analysis was found within the acceptable limits of the calculated values (Micro-analytical Unit, NRC). Infrared spectra (KBr) were recorded on a Nexus 670 FTIR Nicolet, Fourier Transform infrared spectrometer (Perkin Elmer, Hopkinton, MA, USA**). Proton nuclear magnetic resonance (1H-NMR) spectra were run in [d6] DMSO on Jeol 270 MHz or 500 MHz instruments (Joel Inc., Tokyo, Japan). Chemical shifts d are given in ppm. Mass spectra were run on a MAT Finnigan SSQ 7000 spectrometer (Shimadzu, Kyoto, Japan; model: QP2010 ultra), using the electron impact technique (EI). Analytical thin layer chromatography (TLC) was performed on silica gel aluminium sheets, 60 F254 (E. Merck**). The following solvent systems (by volume) were used as eluents for the development of the plates: S: chloroform–methanol–acetic acid (85:10:5); S1: S-petroleum ether (b.p. 40–60^ ^°C) (1:1); S2: S-petroleum ether (b.p. 40–60 °^ ^C) (3:2); S3: S-petroleum ether (b.p. 40–60 ^ ^°C) (1:2) and S4: butanol–water–acetic acid–pyridine (120:48:12:40). It is generally known that basic reaction media enhance racemization. However, under the reaction conditions employed in this work, especially short reaction times and temperatures below (°C), only negligible racemization was observed.

#### 2.1.1. Synthesis of l-Alanine methyl ester, isophthaloyl chloride (2)

The titled compound (**2**) prepared according to previously reported methods^29–31^.

#### 2.1.2. Synthesis of isophthaloyl-bis-[l-Alanine methyl ester] (3)

A dichloromethane (DCM) solution of free l-Alanine methyl ester (30 mmol, –20 °C) was added to a DCM solution of the isophthaloyl chloride **(2)** (–20 °C, 15 mmol). And then the reacted mixture was stirred for (3 h, –20 °C), then for (24 h at room temperature). It was then washed with (water, 1 N sodium bicarbonate and 1 N potassium hydrogen sulphate), followed by water again and then dried over sodium sulphate. Next, the solvent was evaporated and the obtained compound was solidified by petroleum ether (boiling point 40–60 °C). The solid was filtered off, dissolved in methanol (MeOH) and precipitated by petroleum ether to give compound **3.**

Dimethyl 2, 2'-(isophthaloyl-*bis*-(azanediyl)) dipropionate (**3**):

*Yield:* 75%; *melting point:* 132–134 °C, *IR (cm^−1^): (KBr): ν* = 3330 (NH stretching), 3060 (CH, aromatic), 2950 (CH, aliphatic), 1744 (C = O, ester), 1675, and 1610 (C = O amide I and II, respectively).*^1^H-NMR (500 MHz, δ, ppm, DMSO-d_6_): δ* = 8.90, 8.85 (s, 2H, 2NH, D_2_O exchangeable, amide), 8.50–8.30 (4H, aromatic H), 4.35–4.25 (m, 2H, 2CH, α-l-Ala), 3.70–3.50 (s, 6H, 2OCH_3_), 1.50–1.44 (d, 6H, 2CH_3_, β-l-Ala). *MS (EI, 70* eV)*: m/z (%) =* 336 (M^+^, 6.11), 222 (3.06), **78 (100)**, 63*. Molecular formula (molecular weight):* C_16_H_20_N_2_O_6_ (336.3). *Calculated analysis:* C, 57.14; H, 5.99; N, 8.33; *Found:* C, 57.10; H, 5.90; N, 8.8.

#### 2.1.3. Synthesis of N^1^,N^3^-bis-(1-hydrazinyl-1-oxopropan-2-yl) isophthalamide (4)

To a stirred methanolic solution (50 mL) of the corresponding amino acid methyl ester (**3**) (1 mmol), hydrazine hydrate 99% (0.35 mL, 10 mmol) was added. The mixture was then refluxed for 3 h, the solvent was evaporated and the obtained residue was triturated with diethylether, filtered off, and precipitated from MeOH/diethylether to give **4**.

*Yield:* 75%; *melting point:* 232–234 °C, *IR (cm^−1^): (KBr):ν* = 3293 (2NH_2_), 3131 (4NH, amide), 2972 (CH aliphatic), 1650 (4CO), 1528 (2 C = N). *^1^H-NMR (500 MHz, δ, ppm, DMSO-d_6_): δ* = 1.36–1.49 (m, 6H, 2CH_3_), 4.24 (s, 4H, 2NH_2_), 4.57, 5.38 (2q, 2H, 2CH–CH_3_), 7.24–8.25 (m, 4H, Ar-H), 8.74, 9.70 (s, 4H, 4NH). *MS (EI, 70 eV): m/z (%) =* 336 (M^+^, 16), 331 (10), 323 (25), 316 (23), 310 (35), 304 (69), 300 (25), 299 (15), 285 (24), 281 (19), 277 (11), 266 (21), 258 (31), 124 (13), 93 (12), 77 (18), **42 (100)**. *Molecular formula (molecular weight):* C_14_H_20_N_6_O_4_ (336.3). *Calculated analysis:* C, 49.99; H, 5.99; N, 24.99; *Found:* C, 49.92; H, 5.97; N, 24.88.

#### 2.1.4. Synthesis of N^1^-(1-oxo-1-(-2-(2-oxoindolin-3-ylidene) hydrazinyl) propan-2-yl)-N^3^-(1-oxo-1-(-2-(2-oxoindolin-3-ylidene) hydrazinyl) propan-2-yl) isophthalamide (***5***) and N^1^-(1-(-2-(5-chloro-2-oxoindolin-3-ylidene)hydrazinyl)-1-oxopropan-2-yl)-N^3^-(1-(-2-(5-chloro-2-oxoindolin-3-ylidene)hydrazinyl)-1-oxopropan-2-yl)isophthalamide (***6****)*

To a solution of compound *bis* (1-hydrazinyl-1-oxopropan-2-yl) isophthalamide (**4**) (3.36 g, 0.01 mol) in DMF (10 ml) was added isatin and/or chloroisatin (0.2 mol) refluxed for 4 h, cold poured on to crushed ice. The product formed was filtered off and crystallized from ethanol to give the target compounds.

*N^1^-(1-oxo-1-(-2-(2-oxoindolin-3-ylidene) hydrazinyl) propan-2-yl)-N^3^-(1-oxo-1-(-2-(2-oxoindolin-3-ylidene) hydrazinyl) propan-2-yl) isophthalamide (****5****)*

*Yield:* 75%; *melting point:* 271–273 °C, *IR (cm^−1^): (KBr): ν* = 3391, 3235 (6NH), 2923 (CH aliphatic), 1698, 1647 (4CO), 1524 (2 C = N). *^1^H-NMR (500 MHz, δ, ppm, DMSO-d_6_): δ* = 1.49–1.51 (m, 6H, 2CH_3_), 4.64, (m, 2H, CHCH_3_), 6.92–8.49 (m, 10H, Ar-H), 10.00 (s, 2H, NH, pyrrole ring), 11.25, 13.50 (s, 4H, 4NH). *MS (EI, 70 eV): m/z (%) =* 594 (M^+^, 6), 531 (11), 487 (13), 419 (16), 389 (17), 371 (27), 362 (13), 346 (11), 317 (22), 308 (13), 288 (21), 160 (98), 143 (16), 132 (43), 129 (39), 118 (20), **103 (100)**, 88 (23), 76 (74), 62 (28). *Molecular formula (molecular weight):* C_30_H_26_N_8_O_6_ (594.5). *Calculated analysis:* C, 60.60; H, 4.41; N, 18.85; *Found:* C, 60.64; H, 4.45; N, 18.82.

*N^1^-(1-(-2-(5-chloro-2-oxoindolin-3-ylidene)hydrazinyl)-1-oxopropan-2-yl)-N^3^-(1-(-2-(5-chloro-2-oxoindolin-3-ylidene) hydrazinyl)-1-oxopropan-2-yl)isophthalamide (****6****)*

*Yield:* 70%; *melting point:* 271–273 °C, *IR (cm^−1^): (KBr): ν* = 3393, 3239 (6NH), 2922 (CH aliphatic), 1698 (2CO) 1647 (2CO, amide), 1521 (2 C = N). *^1^H-NMR (500 MHz, δ, ppm, DMSO-d_6_): δ* = 1.49–1.53 (m, 6H, 2CH_3_), 4.65 (m, 2H, CHCH_3_), 6.95–8.59 (m, 10H, Ar-H), 10.30 (s, 2H, NH, pyrrole ring), 11.35, 13.45 (s, 4H, 4NH). *MS (EI, 70 eV): m/z (%) =* 663 (M^+^, 12), 637 (19), 586 (24), 486 (21), 446 (83), 433 (31), 418 (48), 413 (16), 401 (21), 387 (24), 378 (58), 194 (52), 142 (13), 132 (14), 116 (18), 109 (20), **85 (100).** *Molecular formula (molecular weight):* C_30_H_24_Cl_2_N_8_O_6_: (663.47). *Calculated analysis:* C, 54.31; H, 3.65; Cl, 10.69; N, 16.89; *Found:* C, 54.36; H, 3.59; Cl, 10.61; N, 16.79.

#### 2.1.5. Synthesis of N^1^,N^3^-bis(1-(5-thioxo-4,5-dihydro-1,3,4-oxadiazol-2-yl)ethyl)isophthalamide (***7****)*

A mixture of N^1^, N^3^-*bis* (1-hydrazinyl-1-oxopropan-2-yl) isophthalamide (3.3 g, 0.01 mol) and carbon disulphide (6 ml) in pyridine (10 ml) and DMF (5 ml) was heated under reflux on water bath for 8 h. After cooling, the solvent was evaporated under reduced pressure and residue was triturated with an ice-water mixture and neutralized with diluted HCl. the solid precipitate formed was filtered off and recrystallized from ethanol to afford **7** as pale yellow crystals.

*N^1^,N^3^-bis (1-(5-thioxo-4,5-dihydro-1, 3, 4-oxadiazol-2-yl) ethyl) isophthalamide (****7****)*

*Yield:* 80%; *melting point:* 132–134 °C, *IR (cm^−1^): (KBr): ν* = 3428, 3290 (4NH), 2919 (CH, aliphatic), 1635 (2CO), 1520 (2 C = N), 1114, 1057 (2 C = S). *^1^H-NMR (500 MHz, δ, ppm, DMSO-d_6_): δ* = 1.17–1.41 (m, 6H, 2CH_3_), 3.35–4.14 (m, 2H, CHCH_3_), 7.71–8.39 (m, 4H, Ar-H), 873, 9.25 (s, 4H, 4NH), 7.00 (s, 2H, NH, oxadiazole ring). *MS (EI, 70 eV): m/z (%) =* 420 (M^+^, 5.5), 407 (10), 393 (10), 392 (16), 97 (19), **70 (100)**, 57 (59). *Molecular formula (molecular weight):* C_16_H_16_N_6_O_4_S_2_: (420.07). *Calculated analysis:* C, 45.71; H, 3.84; N, 19.99; S, 15.25; *Found:* C, 45.75; H, 3.82; N, 19.89; S, 15.28.

#### 2.1.6. General procedure for the synthesis of sugar hydrazone (8, 9)

A mixture of N^1^,N^3^-*bis*(1-hydrazinyl-1-oxopropan-2-yl) isophthalamide (3.3 g, 0.01 mol) in absolute ethanol (20 ml) containing glacial acetic acid, the respective monosaccharide(3 g, 0.02 mol) in water (2 ml). The mixture was heated under reflux for 3 h and the resulting solution was concentrated and left to cool to room temperature. The formed precipitate was filtered off, washed with water and ethanol, then dried and recrystallized from ethanol to afford **8** and **9** as white crystals.

*N^1^,N^3^-bis (1-oxo-1-(-2-((2S, 3 R, 4 R)-2, 3, 4, 5-tetrahydroxypentylidene) hydrazinyl) propan-2-yl) isophthalamide (****8****)*

*Yield:* 60%; *melting point:* <300 °C, *IR (cm^−1^): (KBr):ν* = 3427 (OH), 3365, 3307 (2NH), 2922 (CH aliphatic), 1639 (4CO), 1527 (2 C = N). *^1^H-NMR (500 MHz, δ, ppm, DMSO-d_6_): δ* = 1.17–1.41 (m,6H, 2CH_3_), 3.35 (m, 10H, H-5,5′, H-4,3,2), 4.11 (m, 2H, 2OH), 4.45 (m, 4H, 4OH), 4.59 (m, 2H, CHCH_3_), 5.30 (m, 2H, 2OH), 7.62–8.52 (m, 6H, Ar-H + H1),839, 8.80 (4H, 4NH). *Molecular formula (molecular weight):* C_25_H_37_N_5_O_12_: (599.59). *Calculated analysis:* C, 50.08; H, 6.22; N, 11.69; *Found:* C, 50.14; H, 6.27; N, 11.54.

*N^1^,N^3^-bis (1-oxo-1-(-2-((2R, 3S, 4R)-2, 3, 4, 5-tetrahydroxypentylidene) hydrazinyl) propan-2-yl) isophthalamide (****9****):*

*Yield:* 60%; *melting point:* < 300 °C, *IR (cm^−1^): (KBr):ν* = 3423 (OH),(3360, 3320 (2NH), 2922 (CH aliphatic), 1645 (4CO), 1531 (2 C = N). *^1^H-NMR (500 MHz, δ, ppm, DMSO-d_6_): δ* = 1.18–1.42 (m,6H, 2CH_3_),3.78 (m, 10H, H-5,5′, H-4,3,2), 4.13 (m, 2H, 2OH), 4.45–4.52 (m, 4H, 4OH), 4.86 (m, 2H, CHCH_3_), 5.31 (m, 2H, 2OH), 7.62–8.52 (m, 6H, Ar-H + H1), 872,8.83 (4H, 4NH). *Molecular formula (molecular weight):* C_25_H_37_N_5_O_12_: (599.59). *Calculated analysis:* C, 50.08; H, 6.22; N, 11.69; *Found:* C, 50.18; H, 6.24; N, 11.66.

#### *2.1.7. General procedure for preparation of Schiff's base compounds (10*–*13)*

A solution of *bis* hydrazide compound (3.36 g,0.01 mol) in absolute ethanol (20 ml) containing glacial acetic acid (5 ml) was added different aromatic aldehydes (0.02 mol) the reaction mixture was refluxed for 6–8 h. The formed precipitate was filtered off and crystallized form acetic acid to afford compounds **10**–**13**.

*N^1^,N^3^-bis-(1-(-2-(4-chlorobenzylidene) hydrazinyl)-1-oxopropan-2-yl) isophthalamide (****10****):*

*Yield:* 80%; *melting point:* 296–298 °C, *IR (cm^−1^): (KBr): ν* = 3332, 3201 (4NH), 2983 (CH, aliphatic), 1639 (2CO), 1537 (2 C = N). *^1^H-NMR (500 MHz, δ, ppm, DMSO-d_6_): δ* = 1.34–1.45 (m, 6H, 2CH_3_), 4.55, 5.34 (2q, 2H, 2CH–CH_3_), 7.23–8.25 (m, 12H, Ar-H), 8.50 (s, 2H, –6.92–8.49 (m, 10H, Ar-H), 10.00 N = CH), 8.72, 8.81 (2d, 2H, NH). 11.31, 11.40 (2 s, 2H, NH). *MS (EI, 70 eV): m/z (%) =* 581 (M^+^, 5), 575 (11), 523 (26), 519 (10), 494 (14), 488 (28), 460 (17), 425 (14), 434 (31), 421 (40), 418 (15), 401 (24), 390 (39), 368 (100**)**, 365 (21), 350 (31), 348 (15), 336 (16), 293 (12), 133 (10), 95 (20).*Molecular formula (molecular weight):* C_28_H_26_Cl_2_N_2_O_4_ (581.14). *Calculated analysis:* C, 57.84; H, 4.51; Cl, 12.19; N, 14.45; *Found:* C, 57.83; H, 4.52; Cl, 12.09; N, 14.47.

*N^1^,N^3^-bis-(1-(-2-(4-fluorobenzylidene) hydrazinyl)-1-oxopropan-2-yl) isophthalamide (****11****)*

*Yield:* 85%; *melting point:* 285–287 °C, *IR (cm^−1^): (KBr): ν* = 3332, 3213 (4NH), 2981 (CH, aliphatic), 1645 (2CO), 1537 (2 C = N). *^1^H-NMR (500 MHz, δ, ppm, DMSO-d_6_): δ* = 1.37–1.47 (m, 6H, 2CH_3_), 4.56, 5.32 (2q, 2H, 2CH–CH_3_), 7.27–8.27 (m, 12H, Ar-H), 8.55 (s, 2H, –N = CH), 8.74, 8.82 (2d, 2H, 2NH). 11.30, 11.42 (s, 2H, 2NH). *MS (EI, 70 eV): m/z (%) =* 548 (M^+^, 6.4), 516 (20.5), 487 (10), 477 (37.9), 463 (11.2), 433 (42), 419 (13), 406 (53), 394 (17), 394 (17), 376 (24), 365 (75), 339 (100**)**, 321 (59), 147 (19), 74 (63). *Molecular formula (molecular weight):* C_28_H_26_F_2_N_6_O_4_ (548.55). *Calculated analysis:* C, 61.31; H, 4.78; F; 6.93, N, 15.32; *Found:* C, 61.32; H, 4.76; F; 6.95, N, 15.34.

*N^1^,N^3^-bis-(1-(-2-(4-methoxybenzylidene) hydrazinyl)-1-oxopropan-2-yl) isophthalamide (****12****)*

*Yield:* 80%; *melting point:* 273–275 °C, *IR (cm^−1^): (KBr): ν* = 3332, 3205 (4NH), 2981 (CH, aliphatic), 1641 (2CO), 1533 (2 C = N). *^1^H-NMR (500 MHz, δ, ppm, DMSO-d_6_): δ* = 1.43–1.47 (m, 6H, 2CH_3_), 3.08 (s, 6H, 2OCH_3_), 4.56, 5.34 (2q, 2H, 2CHCH_3_), 7.00–8.21 (m, 12H, Ar-H), 8.45 (s, 2H, -N = CH), 8.69, 8.78 (2d, 2H, 2NH), 11.30, 11.46 (2 s, 2H, 2NH). *MS (EI, 70 eV): m/z (%) =* 572 (M^+^, 19%), 544 (28), 504 (25), 496 (19), 487 (22), 462 (16), 431 (36), 419 (25), 395 (33), 389 (22), 347 (24), 331 (24), 312 (100**)**, 295 (34), 84 (64), 59 (77). *Molecular formula (molecular weight):* C_30_H_32_N_6_O_6_ (572.62). *Calculated analysis:* C, 62.93; H, 5.63; N, 14.93; *Found:* C, 62.95; H, 5.64; N, 14.98.

*N^1^,N^3^-bis-(1-(-2-(3, 4-dimethoxybenzylidene) hydrazinyl)-1-oxopropan-2-yl) isophthalamide (****13****)*

*Yield:* 85%; *melting point:* 277–279 °C, *IR (cm^−1^): (KBr): ν* = 3330, 3215 (4NH), 2985 (CH, aliphatic), 1647 (2CO), 1536 (2 C = N). *^1^H-NMR (500 MHz, δ, ppm, DMSO-d_6_): δ* = 1.43–1.46 (m, 6H, 2CH_3_), 3.08 (s, 12H, 4OCH_3_), 4.55, 5.35 (2q, 2H, 2CHCH_3_), 7.00–8.42 (m, 10H, Ar-H), 8.50 (s, 2H, –N = CH), 8.71,8.74 (2d, 2H, 2NH),11.32, 11.467 (2 s, 2H, 2NH). *MS (EI, 70 eV): m/z (%) =* 632 (M^+^, 9.4), 607 (19), 592 (10), 582 (16), 54713), 558 (21), 547 (9), 493 (13), 488 (32), 462 (35), 390 (24), 318 (16), 300 (100**)**, 206 (17), 76 (10), 40 (18)*. Molecular formula (molecular weight):* C_32_H_32_N_6_O_8_ (632.26). *Calculated analysis:* C, 60.75; H, 5.74; N, 13.28; *Found:* C, 60.74; H, 5.76; N, 13.38.

#### 2.1.8. General procedure for preparation of oxathiazolidin compounds (14–16)

Thioglycolic acid (0.01 mol) was added to a well stirred solution of Schiff’s bases **10**–**12** (0.01 mol) in dry benzene (20 ml), and then refluxed for 7–9 h. After completion of the reaction, excess solvent was evaporated under reduced pressure and the residue was neutralized with cold dilute sodium bicarbonate solution, the formed product was filtered off, washed with water and then re-crystallized from methanol to give compounds **14**–**16**.

*N^1^,N^3^-bis-(1-(2-(4-chlorophenyl)-4-oxothiazolidin-3-ylamino)-1-oxopropan-2-yl) isophthalamide (****14****)*

*Yield:* 65%; *melting point:* 218–220 °C, *IR (cm^−1^): (KBr): ν* = 3424, 3345 (4NH), 2925 (CH, aliphatic), 1724, 1677 (4CO). *^1^H-NMR (500 MHz, δ, ppm, DMSO-d_6_): δ* = 1.15, 1.29 (2d, 6H, 2CH_3_), 3.70, 3.83 (s, 2H, S-CH_2,_ thiazolidin ring), 4.42 (2 m, 2H, 2CH-CH_3_), 5.80, 5.93 (2 s, 2H, 2 N-CH), 7.15–7.97 (m, 12H, Ar-H), 8.33, 8.75 (2d, 2H, 2NH). 10.29 (s, 2H, 2NH). *MS (EI, 70 eV): m/z (%) =* 731 (M^+^+1, 0.23), 730 (M^+^, 1), 385 (1.4), 355 (9), 314 (2.5), 286 (2.5), 245 (11), 229 (7), 212 (22), 202 (14), 174 (23), 166 (13), 158 (15), 148 (18), 130 (17), 111 (30), 102 (18), 97 (100**),** 71 (53), 67 (16)*. Molecular formula (molecular weight):* C_32_H_30_Cl_2_N_6_O_6_S_2_ (729.65). *Calculated analysis:* C, 52.68; H, 4.14; Cl, 52.68 N, 11.52; S, 8.79; *Found:* C, 52.69; H, 4.16; Cl, 52.69 N, 11.57; S, 8.76.

*N^1^,N^3^-bis-(1-(2-(4-fluorophenyl)-4-oxothiazolidin-3-ylamino)-1-oxopropan-2-yl) isophthalamide (****15****)*

*Yield:* 64%; *melting point:* 168–170 °C, *IR (cm^−1^): (KBr): ν* = 3423, 3340 (4NH), 2921 (CH, aliphatic), 1721, 1679 (4CO). *^1^H-NMR (500 MHz, δ, ppm, DMSO-d_6_): δ* = 1.13, 1.28 (2d, 6H, 2CH_3_), 3.69, 3.85 (s, 2H, S-CH_2,_ thiazolidin ring), 4.42 (2 m, 2H, 2CH-CH_3_), 5.80, 5.93 (2 s, 2H, 2 N-CH), 7.15–7.96 (m, 12H, Ar-H), 8.31,8.73 (2d, 2H,2 NH),10.27 (s, 2H, 2NH).*^13^C-NMR (125 MHz, δ, ppm, DMSO-d_6_): δ* = 19.32 (2 CH_3_), 29.77 (2S-CH_2_), 49.75 (2 CH-CH_3_), 61.60 (N-CH), 115.69–158.00 (Ar-C), 166.03, 168.00, 172.14 (3 C = O).*MS (EI, 70 eV): m/z (%) =* 696 (M^+^, 3.7), 601 (4), 578 (8), 461 (5.6), 414 (21), 382 (10), 366 (13), 351 (28), 333 (28), 324 (23), 294 (39), 285 (51), 281 (95), 267 (19), 256 (23), 130 (4), 80 (10), 78 (22), 56 (18), 40 (100). *Molecular formula (molecular weight):* C_32_H_30_N_6_O_6_S_2_ (696.16). *Calculated analysis:* C, 55.16; H, 4.34; F, 5.45; N, 12.06; S, 9.20; *Found:* C, 55.19; H, 4.36; F, 5.49 N, 12.16; S, 9.25.

*N^1^,N^3^-bis -(1-(2-(4-methoxyphenyl)-4-oxothiazolidin-3-ylamino)-1-oxopropan-2-yl) isophthalamide* ***(16)***

*Yield:* 65%; *melting point:* 242–244 °C, *IR (cm^−1^): (KBr): ν* = 3424, 3338 (4NH), 2923 (CH, aliphatic), 1725.1678 (4CO). *^1^H-NMR (500 MHz, δ, ppm, DMSO-d_6_): δ* = 1.16, 1.30 (2d, 6H, 2CH_3_), 3.72, 3.76 (2 s, 6H, 2OCH_3_), 3.84–3.88 (s, 2H, S-CH_2,_ thiazolidin ring), 4.46, 4.52 (2 M, 2H, 2CHCH_3_), 5.72, 5.79 (2 s, 2H, 2N-CH) 7.33–8.23 (m, 12H, Ar-H), 8.061, 8.68 (2d, 2H, 2NH). 1023 (s, 2H, 2NH). *MS (EI, 70 eV): m/z (%) =* 721 (M^+^+1, 2.3), 721 (M^+^, 9.8), 710 (19), 687 (11), 659 (12), 650 (27), 627 (20), 593 (16), 589 (45), 564 (34), 535 (41), 503 (52), 477 (100**),** 465 (16), 422 (25), 381 (30), 353 (12), 336 (17), 236 (11), 97 (25), 73 (29). *Molecular formula (molecular weight):* C_34_H_36_N_6_O_8_S_2_ (720.20). *Calculated analysis:* C, 56.65; H, 5.03; N, 11.66; S, 8.90; *Found:* C, 56.62; H, 5.05; N, 11.68; S, 8.92.

### 2.2. Biological evaluations

#### 2.2.1. *In vitro* cytotoxic activity

Human colon carcinoma (HCT-116 cell line), human lung carcinoma (A-549), human breast carcinoma (MCF-7 cell line) and human skin normal cell line (BJ-1) were obtained from Karolinska Center, Department of Oncology and Pathology, Karolinska Institute and Hospital, Stockholm, Sweden. IC_50_ values were performed using SPSS computer program (SPSS for windows, statistical analysis software package/version 9/1989 SPSS Inc., Chicago, IL).

The procedure was done in laminar air flow cabinet bio safety class II level. Culturing and sub culturing were carried out according to Thabrew et al.^32^. Doxorubicin was used as a positive control. DMSO used as negative control. Cell Viability Assay was done according to Selim et al.^33^ as described by Mosmann et al.^34^. The cells were seeded at concentration of 10 × 10^3^ cells per well in case of MCF-7, 20 × 10^3^ cells/well in case of HCT-116 cell lines using 96-well plates at 37 °C. After 48-h incubation, the medium was aspirated and 40 μl MTT salt (2.5 mg/ml) were added and further incubated for 4 h. About 200 μl 10% sodium dodecyl sulphate (SDS) was added. The absorbance was measured at 595 nm.

#### 2.2.2. *In vitro* enzyme inhibition assay

The *in vitro* enzyme inhibition assessment for compound **5** was performed at confirmatory diagnostic unit, Vacsera, Egypt. The screening achieved profiling of the compound **5** against a range of four protein kinases [EGFR, VEGFR-2, CDK-2 and c-kit] by ELISA assay method using staurosporine as a standard according to the previously reported methods^28^.

#### 2.2.3. Effect of compound 5 on the level of Bax/BCL-2/p53

Bax protein levels were evaluated according to Onur et al. ^35^. Monoclonal antibody specific to Bax captured on the plate is added. After incubation, Strep avid in conjugated to Horseradish peroxidase (HRP) is added. The reaction is terminated by the addition of acid and optical density of the colour produced measured at 450 nm.

BCL-2 in the samples and standards were estimated according to Barbareschi et al. ^36^. A biotin-conjugated antibody was added followed by streptavidin-HRP. The reaction is then terminated by addition of acid and absorbance was measured at 450 nm.

Human p53 present in the sample or standard binds to antibodies adsorbed to the microwells. A biotin-conjugated is added. After incubation and dispense of unbound biotin-conjugated streptavidin HRP is added. The reaction is terminated by addition of acid and absorbance is measured at 450 nm^37^.

#### 2.2.4. Human caspase-7 (CASP-7) estimation

The micro ELISA plate provided in this kit pre-coated with CASP7-specific antibody. A biotinylated CASP7 antibody and Avidin–HRP conjugate was added. Aspire the excess components. The substrate solution was added wells that contain CASP7, biotinylated detection antibody and Avidin–HRP conjugate will appear blue in colour. The colour turns yellow followed the addition of sulphuric acid solution. The optical density (OD) was measured at a wavelength of 450 nm ± 2 nm^38^.

#### 2.2.5. Enzyme-linked immunosorbent assay kit for tubulin beta (TUBb)

MCF-7 cells were inoculated at concentration 1.2–1.8 × 10,000 cells/well using DMEM (supplemented with 10% FBS and 1% penicillin–streptomycin). Tested compound added for 18–24 h before the enzyme assay for Tubulin. Avidin conjugated to HRP is added to each microplate well and incubated. After TMB substrate solution is added, wells that contain TUBb, biotin-conjugated antibody and enzyme-conjugated Avidin will exhibit a change in colour. Sulphuric acid solution added to terminate enzymatic reaction. Colour change is measured spectrophotometrically at a wavelength of 450 nm ± 10 nm^39^.

#### 2.2.6. Measurement of DNA fragmentation using DPA assay

DNA fragmentation of the cells was assayed, as previously described^40^^,^^41^. Briefly, the cells were lysed with 20 mM EDTA, 0.5% (v/v) Triton X-100, and 5 µM Tris (pH 8.0) for 15 min on ice. The cells were then centrifuged for 20 min, at 27,000×*g*, to separate intact chromatin from DNA fragments. The amount of DNA was measured by using a diphenylamine reagent. The optical density is measured at 600 nm.

### 2.3. Molecular docking studies

The molecular modelling of the compound **5** was carried out using molecular operating environment (MOE, 10.2008) software^42^. The X-ray crystallographic structure of EGFR co-crystallized with erlotinib as an inhibitor (PDB ID: 1M17)^43^ was downloaded from the protein data bank. The receptor was prepared for docking study using Protonate 3D protocol in MOE with default options followed by water molecules removal. The co-crystalized ligand was used to define the active site for docking. Docking setup was first validated by re-docking of the co-crystallized ligand in the vicinity of the active site of the receptor. The validated setup was then used in predicting the ligand–receptor interactions at the active site for compound **5**.

## 3. Results and discussion

### 3.1. Chemistry

**Scheme 1.**
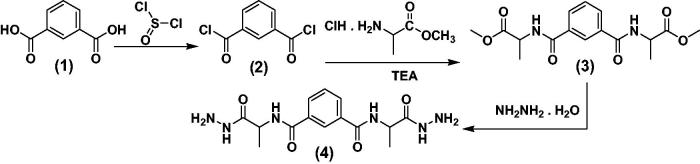
Synthetic routes for N^1^,N^3^-bis-(1-hydrazinyl-1-oxopropan-2-yl) isophthalamide (**4**).

In our previous studies of amino acid and peptide derivatives, gave a good antimicrobial properties, anti-inflammatory, analgesic agents^44–48^ and anticancer activities^26–28^^,^^49^, as well as, pyrazoloderivatives as anticancer agents^50^. Thus, our study aims to conjugate l-alanine and isophthalic acid. The novel candidates **3**–**16** were synthesized based on N^1^,N^3^-bis-(1-hydrazinyl-1-oxopropan-2-yl) isophthalamide (**4**) which may be expected to possess promising anticancer properties. Accordingly, a rational design, synthesis and structural characterization of N^1^, N^3^-*bis* (1-hydrazinyl-1-oxopropan-2-yl) isophthalamide, were synthesized *via* synthetic peptide coupling methods, (in solution). The compound N^1^,N^3^-*bis*-(1-hydrazinyl-1-oxopropan-2-yl) isophthalamide (**4**) was synthesized by the reaction of isophthalic acid (**1**) with thionyl chloride, to give isophthaloyl chloride (**2**), while compound **2** was then coupled with a free l-Alanine methyl ester to give dimethyl 2,2'-(isophthaloyl-*bis*-(azanediyl)) dipropionate (**3**). IR of compound **3** showed absorption bands at 3330 cm^−1^ due to NH in addition to absorptions of carbonyl groups at 1744 cm^−1^ (C = O ester). ^1^H-NMR of ester **3** revealed two signals in the region *δ* 3.7–3.5 of 2CH_3_(CH_3_ ester) in addition to the D_2_O exchangeable signals of amidic. Hydrazinolysis of dimethyl 2,2'-(isophthaloyl-*bis*-(azanediyl)) dipropionate (**3**) with hydrazine hydrate led to the corresponding hydrazide **4** in 75% yield (Scheme 1). ^1^H-NMR of compound **4** exhibited the D_2_O exchangeable signals at *δ* 9.70 and 8.74 in addition to the signals of 2NH_2_ at *δ* 4.24. The mass spectrum of hydrazide **4** showed a characteristic peaks equal to its molecular weight and base beak at *m/z =* 336 and42, respectively.

**Scheme 2.**
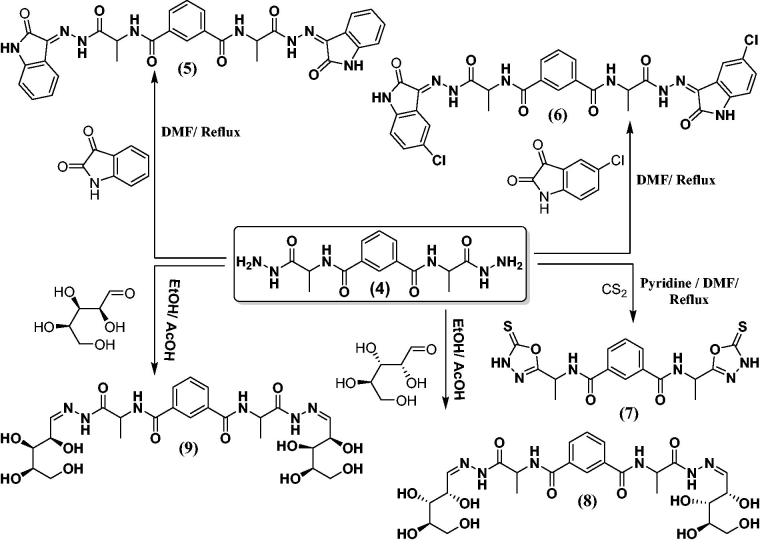
Synthetic routes for N^1^,N^3^- bis-(1-hydrazinyl-1-oxopropan-2-yl) isophthalamide derivatives **5–9**.

The condensation of the *bis* (1-hydrazinyl-1-oxopropan-2-yl) isophthalamide (**4**) with isatin or chloroisatin took place through refluxing in DMF to produced hydrazide substituted candidates **5** and **6** (Scheme 2). ^1^H-NMR of hydrazide candidates **5** and **6** in highly yield (75% and 70%), respectively. showed the characteristic signals of NH, pyrrole ring, at 10.00 and 10.30, respectively, as well as signals of NH in the range *δ* 13.50–11.25. The mass spectrum of hydrazide derivatives **5** and **6** displayed a characteristic peaks equal to its molecular weights at *m/z =* 594 and 663, respectively. On the contrary, hydrazide **4** was reacted with carbon disulphide in pyridine and DMF which produced oxadiazolederivative **7** (Scheme 2). The structure of oxadiazole **7** is established under the basis of their spectral data. In addition to, *N^1^,*N*^3^*-*bis*-(1-hydrazinyl-1-oxopropan-2-yl) isophthalamide (**4**) was reacted with the respective monosaccharide in ethanol containing glacial acetic acid which produced sugar hydrazone derivatives **8** and **9** (Scheme 2). ^1^H-NMR spectra for **8** and **9** exhibited the characteristic signals of CH, –N = CH, at *δ* 7.50, characteristic multiplets at the range *δ* 4.11–5.31 (8 OH of sugar derivatives) and dupletes at *δ* 1.17–1.41 (2CH_3_, l-Ala).

**Scheme 3.**
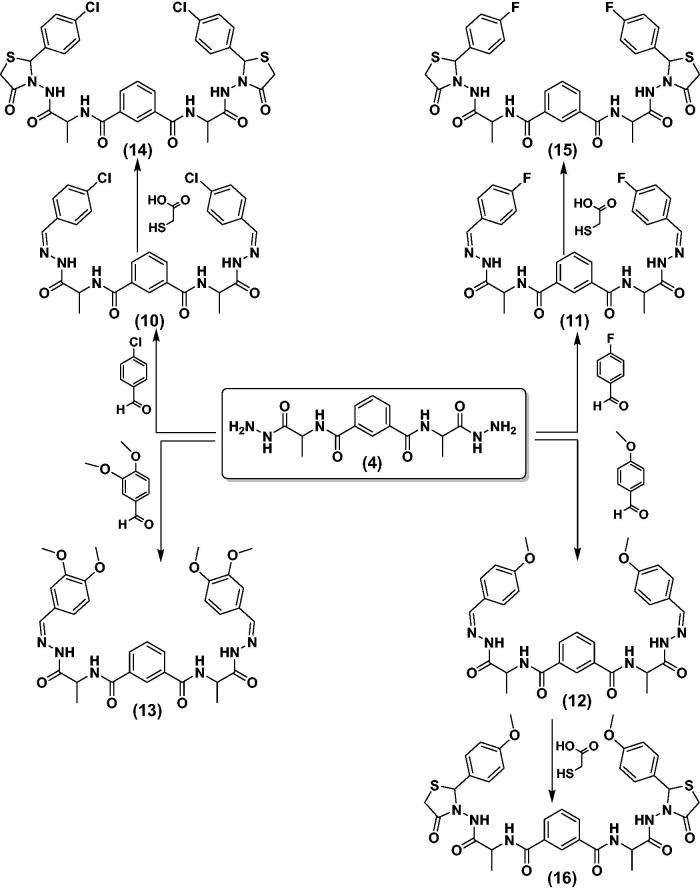
Synthetic routes for N^1^,N^3^-bis-(1-hydrazinyl-1-oxopropan-2-yl) isophthalamide derivatives **10–16**.

The condensation of the hydrazide **4** with 4-chlorobenzaldehyde or 4-fluorobenzaldehyde took place through refluxing in ethanol containing glacial acetic acid. This produced substituted hydrazides **10** and **11** (Scheme 3). ^1^H-NMR of hydrazones **10** and **11** illustrated the characteristic signal of NH in the range *δ* 8.72–8.81, as well as signal of -N = CH at 8.50 and 8.55, respectively. The mass spectrum of **10** and **11** revealed a characteristic peaks equal to its molecular weights at *m/z =* (581 and 548) and base beak at *m/z =* (368 and 339), respectively. On the contrary, *N^1^,N*^3^-*bis* (1-hydrazinyl-1-oxopropan-2-yl) isophthalamide (**4**) was reacted with 4-Anisaldehyde or Veratraldehyde to give the corresponding hydrazide derivatives **12** and **13**, respectively (Scheme 3), and its structures were proven under the basis of their spectral data.

The compounds **10**–**12** were reacted with 2-mercaptoacetic acid in ethanol containing glacial acetic acid to give thiazolidine derivatives **14**–**16**, respectively (Scheme 3). ^1^H-NMR spectra showed characteristic singles at the range *δ* 3.69–3.88 (CH_2_ of thiazolidine ring), 4.42 (2CH of α-l-Ala), and the mass spectrum of **14** and **15** exhibited a characteristic peaks equal to its molecular weights at *m/z =* (731 and 696) and base beak at *m/z =* (97 and 40), respectively.

### 3.2. Biological evaluations

#### 3.2.1. *In vitro* anticancer activity

**Figure 2.**
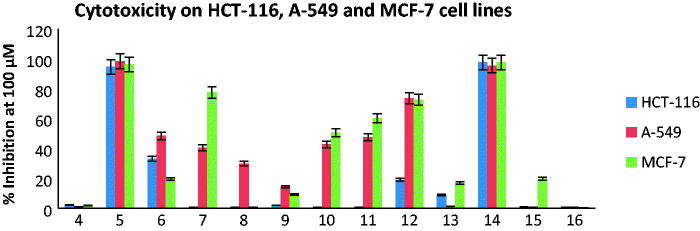
*In vitro* cytotoxicity of compounds **4**–**16**, against human colon tumour (HCT-116), lung tumor (A-549) and human breast tumour (MCF-7) cell lines at concentration 100 µM. Each result is a mean of 3 replicate and values are represented as % inhibition (± standard error).

Cancer is ranked as the second cause global lifelessness; therefore, there is a need for developing an antitumor candidate with minimal side effects to increase the efficacy of chemotherapy. In this study thirteen compounds were preliminary screened for their cytotoxicity utilizing three human carcinoma cell lines, namely human colon carcinoma (HCT-116), lung carcinoma (A-549) and human breast carcinoma (MCF-7) (Figure 2). The compounds which revealed percentage of inhibition higher than 70% were further assessed for determination of their median growth inhibitory concentration (IC_50_) and doxorubicin was used as the reference drug as shown in Table 1. Compounds **5**, **7**, **12** and **14** showed moderate to excellent % of inhibition ranging from 72.5 to 98.3 towards the three tested cancer cell lines.

**Table 1.** IC_50_ values (concentration required to diminish 50% of the cell) of active compounds possessing ≥ 70% cytotoxicity on colon carcinoma (HCT-116), lung carcinoma (A-549) and breast carcinoma (MCF-7) cell lines.

	IC_50_ (mean ± SD) (µM)^a^		
Compd. no.	HCT-116	A-549	MCF-7
**5**	0.014 ± 0.22	0.040 ± 1.43	0.014 ± 0.70
**7**	–	–	0.073 ± 2.91
**12**	–	0.061 ± 2.12	0.051 ± 3.10
**14**	0.054 ± 1.80	0.041 ± 1.91	0.031 ± 1.51
**Doxorubicin**	0.065 ± 1.00	0.049 ± 1.30	0.045 ± 2.20

^a^Values of IC (±standard error) are calculated using SPSS statistical program.

Concerning IC_50_ data, it was observed that only the compounds **5** and **14** exhibited higher potency against all tested cell lines in comparison with the standard drug.

**Figure 3.**
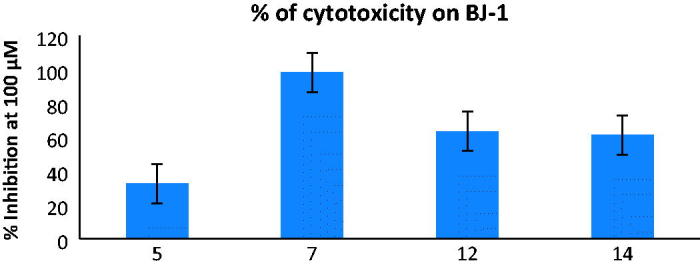
cytotoxic percentage upon normal skin human cell line (BJ-1) at concentration 100 µM of those compounds gave ≥ 70% cytotoxicity over the three tumour cell lines. Each result is a mean of 3 replicate and values are represented as % inhibition (± standard error).

Furthermore, the active compounds **5**, **7**, **12** and **14** were also screened for their cytotoxic effects on human normal skin fibroblasts cell line BJ-1 at 100 μM to test their safety on normal cells (Figure 3). It was clear that the oxoindoline derivative **5** with the promising cytotoxic activity against tested cell lines revealed low cytotoxicity on normal BJ-1 cells with % inhibition 32.6 which makes it interesting candidate for further biological evaluation.

### 3.2.2. Structure–activity relationship

Depending on the previous results, it was noticed that the open chain isophthalamide derivative **4** exhibited very weak cytotoxic activities against HCT-116, A-549 and MCF-7 cell lines (% inhibition = 2.3, 0.9 and 1.8, respectively). Cyclization with 2-oxoindoline moiety gave the most potent derivative **5** against the three tested cell lines (IC_50_ = 0.014 ± 0.22, 0.040 ± 1.43 and 0.014 ± 0.70 µM, respectively) in comparison with the reference drug, doxorubicin (IC_50_ = 0.065 ± 1.00, 0.049 ± 1.30 and 0.045 ± 2.20 µM, respectively). On the one hand, substitution of 2-oxoindoline with chlorine atom at p-5 in compound **6** displayed drastic decrease in the activity towards all tested cell lines (% inhibition = 33.1, 48.4 and 19.6, respectively). On the other hand, cyclization with 1,3,4-oxodiazole scaffold in compound **7** increased the sensitivity only towards MCF-7 cell line (% inhibition = 77.5 and IC_50_ = 0.073 ± 2.91 µM). A dramatic drop in the anticancer effect was detected upon attachment of isophthalamide with 2,3,4,5-tetrahydroxypentyl sugars in **8** and **9**. Insertion of phenyl ring substituted at p-4 with electron withdrawing groups as Cl or F in compounds **10** and **11**, or with electron donating group as methoxy in compound **12** showed variable and noticeable increase in the cytotoxic activity, while substitution with two methoxy groups at p-3,4 led to reduction in the activity due to steric hindrance in compound **13**. The 4-oxothiazolidine derivative linked at p-2 to 4-chlorophenyl **14**, exhibited excellent anticancer potency (IC_50_ = 0.054 ± 1.80, 0.041 ± 1.91and 0.031 ± 1.51 µM, respectively) and it displayed higher percentage of inhibition at 100 μM than the 4-fluorophenyl and 4-methoxyphenyl congeners **15** and **16**.

#### 3.2.3. *In vitro* enzymatic assays

Based on observing the results of *in vitro* cytotoxicity studies of the newly synthesized isophthalamide derivatives, the highly active compound **5** was selected for evaluation of its inhibitory activities versus a panel of four different kinases: epidermal growth factor receptor (EGFR), vascular endothelial growth factor receptor-2 (VEGFR-2), cyclin-dependent kinase-2 (CDK-2) and c-kit (receptor tyrosine kinase type III) using staurosporine as multitarget inhibitor.

From IC_50_ values depicted in Table 2, it was noticed that compound **5** potently inhibited EGFR rather than the reference (IC_50_ = 0.78 ± 1.25 and 1.00 ± 1.12 μM, respectively). In contrast, compound **5** seemed to have weak inhibitory activities against VEGFR-2, CDK-2 and c-Kit kinases with IC_50_ values ranging from 2.85 ± 0.04 to 6.24 ± 1.21 μM.

**Table 2.** Inhibitory evaluation of compound **5** against EGFR, VEGFR-2, CDK-2 and c-Kit kinases.

Kinase	IC_50_ (mean ± SD) (µM)	
	5	Staurosporine
EGFR	0.78 ± 1.25	1.00 ± 1.12
VEGFR-2	3.17 ± 1.00	0.08 ± 1.10
CDK-2	6.24 ± 1.21	0.11 ± 1.13
c-kit	2.85 ± 0.04	0.60 ± 1.00

IC_50_: compound concentration required to inhibit the enzyme activity by 50%, SEM = standard error mean; each value is the mean of three values.

#### 3.2.4. Effect of compound 5 on the level of Bax, BCL-2, p53, CASP-7, tubulin polymerization (TubB) and % of DNA fragmentation:

According to our results, compound **5**, which has the highest activity on all tested cancer cells including breast carcinoma cells, was considered the safest one among other promising compounds on normal skin cells with weak effect of cytotoxicity (32.6%). Therefore, compound **5** was chosen to elucidate its possible apoptotic mode of action on breast carcinoma cells through investigation of its effect on Bax, BCL-2, Bax/BCL2 ratio, P53, CASP-7, Tubulin polymerization (TubB) and % of DNA fragmentation.

**Figure 4.**
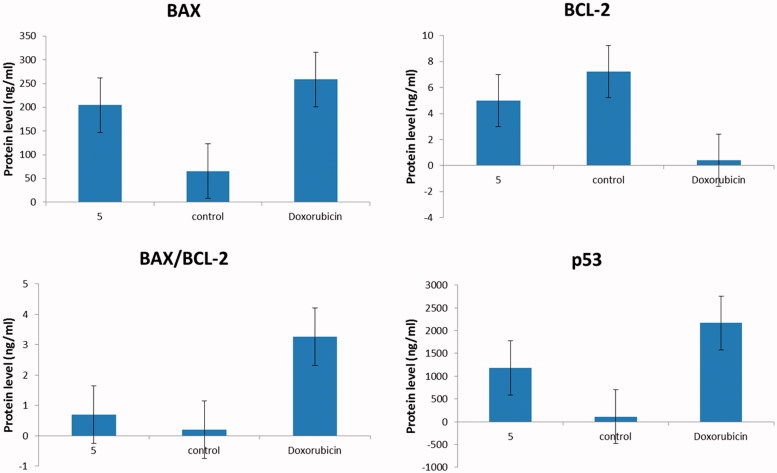
quantitative analysis of Bax, BCL-2 and Bax/BCL-2 ratio and p53 for compound **5** compared to doxorubicin, where *Y*-axis represents the protein levels (ng/ml).

Apoptosis of cancer cells is regulated by the pro-apoptotic Bax, the anti-apoptotic Bcl-2 proteins and the tumour suppressor gene p53^51^. Exposure of MCF-7 cells to compound **5** at IC_50_ for 24 h resulted in significant up-expression of p53 and Bax, with consequent down-expression in the level of BCL-2 compared to the control (Figure 4). As a result, the tested compound **5** showed significant elevation in Bax/BCL-2 ratio, which supports its ability to elevate the therapeutic response in MCF-7 cells.

**Figure 5.**
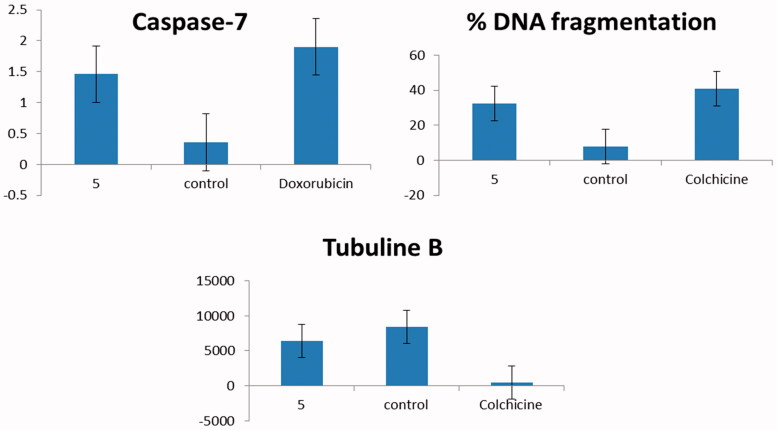
Graphical representation for Caspase-7 concentration (ng/ml) in treated breast cancer cells with compound **5** and untreated compared to doxorubicin and for % DNA fragmentation and IC_50_ (ng/ml) on tubuline B in comparison with colchicine.

There are two vital apoptotic pathways, the death receptor (extrinsic) and the mitochondrial (intrinsic) pathways^52^. The two pathways focus on the same terminal, which was initiated by the activation of *CASP-7* and results in DNA fragmentation^53^. In the current study, compound **5** significantly elevated the Bax/BCL-2 ratio. Therefore, the subsequent step was to investigate the level of active *CASP-7*, which is the key executer of apoptosis. Treatment of MCF-7 cells with compound **5** produced a significant increase in the level of active *CASP-7*. Moreover, compound **5** showed remarkable % of DNA fragmentation and moderate effect on microtubule-polymer mass in comparison with colchicine (Figure 5). In conclusion, compound **5** showed potent pro-apoptotic effect by induction of the intrinsic mitochondrial pathway of apoptosis.

### 3.3. Molecular docking studies

Cytotoxic activity of all synthesized derivatives has been realized by using *in vitro* assay and proved that compound **5** is the most active and promising chemotherapeutic agent. Accordingly, the inhibitory evaluation of compound **5** was carried out against different human proteins which expressed in most of tumour cells especially breast carcinoma namely, EGFR, VEGFR-2, CDK-2 and c-Kit, to validate and specify the mechanism of action or proteins responsible for the anticancer activity.

Consequently, molecular docking was performed with the aim of explaining the promising EGFR inhibitory activity of compound **5** through investigating its binding mode and interaction with the key amino acids (hot spots) in the active site of the EGFR. Docking simulations were done using Molecular Operating Environment software 10.2008 (MOE), Chemical Computing Group Inc., Montreal, Quebec, Canada^42^. The X-ray crystallographic structure of EGFR (pdb code: 1M17)^43^ was downloaded from the protein data bank with its ligand, erlotinib. The docking protocol was validated by re-docking the co-crystallized ligand, erlotinib into the EGFR binding pocket, followed by docking of our compound **5** in the same binding site.

**Figure 6.**
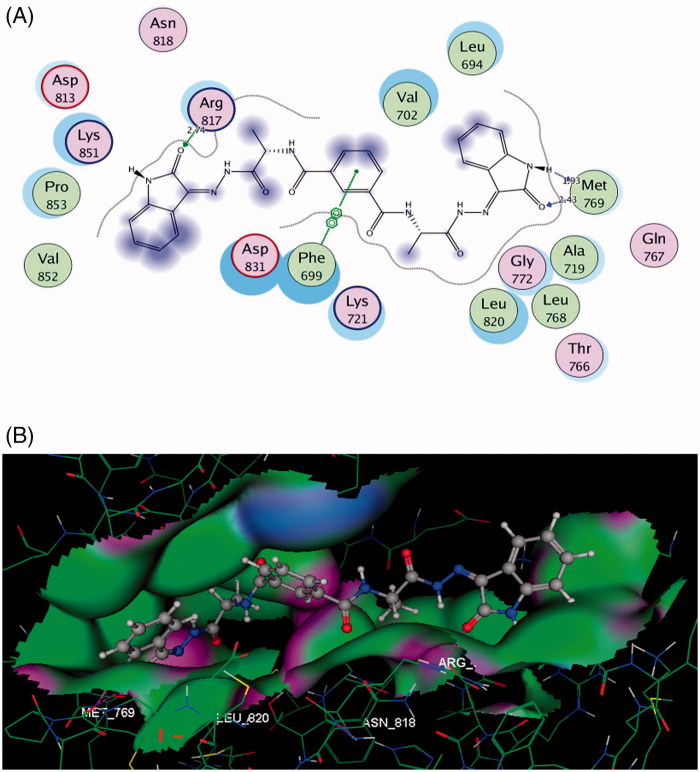
(A and B) Images display 2D and 3D graphs of compound **5** docked into EGFR binding sit (PDB code: 1M17). Green colour indicates hydrophobic area, pink colour indicates high polar area, blue colour indicates mild polar area and dotted lines and arrows represent hydrogen bonds.

The obtained results are pictured in Figures 6 and revealed that the isophthalamide derivative bearing 2-oxoindoline moiety 5, formed two hydrogen bonding with the same amino acid **Met769** as erlotinib, through the N atom and the carbonyl oxygen localized in its oxoindoline moiety (distance: 1.93 and 2.43 Å, respectively). Moreover, the other 2-oxoindoline moiety exhibited a third hydrogen bond acceptor between its carbonyl oxygen and the sidechain of **Arg817** (distance: 2.74 Å). Additionally, a hydrophobic interaction (arene–arene interaction) with EGFR binding site was introduced through the central phenyl moiety of compound **5** and **Phe699**.

This helped us to find an explanation for the excellent binding affinity between compound **5** and EGFR, as presence of two 2-oxoindoline moieties in its structure let it to get good fitting with the binding site.

## 4. Conclusion

In the current study, novel isophthalamide based derivatives incorporated to different hetercycles **4–16** were synthesized using solution phase method in peptide synthesis. All novel compounds were evaluated for their cytotoxic activity upon HCT-116, A-549 and MCF-7 cell lines using MTT assay. Compound **5** exhibited the most significant suppression of the proliferation of the cancer cells. Furthermore, compound **5** was tested against four kinases, and showed significant inhibition of EGFR enzyme. Antitumor potency of compound **5** was further confirmed by studying its effect upon different apoptotic parameters in breast cancer cells. The docking studies also support the results concluded from the enzyme assay and the anti-proliferation screening. It was clear that our ligand **5** that bearing 2-oxoindoline moiety may be considered as a suitable lead for further development of anticancer drugs in future.
